# Posterior Condylar Offset Does Not Correlate With Knee Flexion After TKA

**DOI:** 10.1007/s11999-013-2999-2

**Published:** 2013-04-23

**Authors:** Yoshinori Ishii, Hideo Noguchi, Mitsuhiro Takeda, Junko Sato, Shin-ichi Toyabe

**Affiliations:** 1 Ishii Orthopaedic & Rehabilitation Clinic, 1089 Shimo-Oshi, Gyoda, Saitama 361-0037 Japan; 2 Division of Information Science and Biostatistics, Niigata University Graduate School of Medical and Dental Sciences, Niigata, Japan

## Abstract

**Background:**

Studies of medial and lateral femoral posterior condylar offset have disagreed on whether posterior condylar offset affects maximum knee flexion angle after TKA.

**Questions/purposes:**

We asked whether posterior condylar offset was correlated with knee flexion angle 1 year after surgery in (1) a PCL-retaining meniscal-bearing TKA implant, or in (2) a PCL-substituting mobile-bearing TKA implant.

**Methods:**

Knee flexion angle was examined preoperatively and 12 months postoperatively in 170 patients who underwent primary TKAs to clarify the effect of PCL-retaining (85 knees) and PCL-substituting (85 knees) prostheses on knee flexion angle. A quasirandomized design was used; patients were assigned to receive one or the other implant using chart numbers. A quantitative three-dimensional technique with CT was used to examine individual changes in medial and lateral posterior condylar offsets.

**Results:**

In PCL-retaining meniscal-bearing knees, there were no significant correlations between posterior condylar offset and knee flexion at 1 year. In these knees, the mean (± SD) postoperative differences in medial and lateral posterior condylar offsets were 0.0 ± 3.6 mm and 3.8 ± 3.6 mm, respectively. The postoperative change in maximum knee flexion angle was −5° ± 15°. In PCL-substituting rotating-platform knees, similarly, there were no significant correlations between posterior condylar offset and knee flexion 1 year after surgery. In these knees, the mean postoperative differences in medial and lateral posterior condylar offsets were −0.5 ± 3.3 mm and 3.3 ± 4.2 mm, respectively. The postoperative change in maximum knee flexion angle was −2° ± 18°.

**Conclusions:**

Differences in individual posterior condylar offset with current PCL-retaining or PCL-substituting prostheses did not correlate with changes in knee flexion 1 year after TKA. We should recognize that correctly identifying which condyle affects the results of the TKA may be difficult with conventional radiographic techniques.

**Level of Evidence:**

Level II, therapeutic study. See Guidelines for Authors for a complete description of levels of evidence.

## Introduction

Postoperative maximum knee flexion is a primary functional outcome measure for TKA. Previous studies [1, 3, 11, 12, 18, 19, 23, 24] using radiographic analyses showed contradictory findings regarding whether posterior condylar offset has an effect on knee flexion after TKA. Some studies reported a significant correlation [1, 3, 23, 24], while others reported no correlation [1, 11, 12, 18, 19].

Moreover, the senior author (YI) [16] recently reported that changes in posterior condylar offset based on radiographic evaluations showed no significant correlation with the changes observed in CT evaluated medial and lateral posterior condylar offsets. There are three possible reasons for this discrepancy. First, the femoral condyles are naturally asymmetric in shape and dimension [7]. Second, during TKA, it usually is necessary to remove an asymmetric portion of bone from the posterior femoral condyles to equalize the length of the soft tissue and properly align rotation of the femur, particularly for rectangular flexion gaps [13]. Third, a different magnification of the medial condyle from that of the lateral condyle may be introduced by radiographic evaluations. The magnification of the lateral condyle is always less than that of the medial condyle because the lateral side is always closer to the film plate when taking lateral radiographs or performing videofluoroscopic procedures. Most studies correct for the discrepancy in magnitude between preoperative and postoperative radiographs, but not between the medial and lateral condyles because of the limitations of radiographic evaluations. Ideally, changes in posterior condylar offset should be examined individually for each condyle, as noted by previous studies [1, 3, 23, 24] that found significant correlations between posterior condylar offset and knee flexion angle after TKA. However, correctly recognizing which condyle affects the results of the TKA may be difficult with conventional radiographic techniques. Based on this, CT-based evaluations of medial and lateral posterior condylar offset were recommended when assessing the influence of posterior condylar offset on the knee flexion angle after TKA [16].

To address the question of whether posterior condylar offset has an effect on maximum knee flexion after TKA, it is crucial for surgeons to accurately assess the individual differences in each condyle. A three-dimensional (3-D) lower extremity alignment assessment system [30] (Knee CAS; LEXI, Inc, Tokyo, Japan) has been developed that combines data from computed radiography and CT to enable detection of changes in each condyle. We used this system to try to determine the relationship between posterior condylar offset and knee flexion after TKA with two implant designs.

Specifically, we sought to determine whether posterior condylar offset was correlated with knee flexion angle 1 year after surgery in (1) a PCL-retaining meniscal-bearing TKA implant, or in (2) a PCL-substituting mobile-bearing TKA implant.

## Patients and Methods

The local institutional review board approved this study. All patients provided informed consent. The indication for inclusion in this study was primary osteoarthritis. Contraindications were revision arthroplasties, previous tibial osteotomies, or rheumatoid arthritis. Between January 2006 and December 2011, we performed 175 TKAs in 172 patients. One hundred seventy patients were eligible for inclusion, and all 170 agreed to participate. All knees were implanted with the LCS^®^ Total Knee System (DePuy, Warsaw, IN, USA) (Table 1).

**Table 1:** Patient demographics

Variable	PCL-retaining prosthesis	PCL-sacrificing prosthesis
Number of knees/patients	85/85	85/85
Sex (male/female)	11/74	15/70
Age (years)*	72 (7)	73 (7)
BMI (kg/m^2^)*	26 (4)	27 (4)
Posterior slope (°)*^,†^	10 (2)	10 (2)
Coronal alignment (°)*^,†,‡^	6 (3)	6 (3)
HSS score (points)*	92 (2)	91 (3)

* Values are expressed as mean with SD in parentheses; ^†^evaluated using radiographs; ^‡^valgus; HSS score = Hospital for Special Surgery score of knee function.

Patients had a mean age 73 years (range, 59–86 years) at the time of surgery. Eighty-five knees (85 patients) received meniscal-bearing-type PCL-retaining prostheses, and 85 knees (85 patients) received rotating-platform-type PCL-substituting prostheses. We obtained complete followup data from all patients in this series. Treatment allocation was made using a quasirandomized approach, using even chart numbers for the PCL-retaining group and odd chart numbers for the PCL-substituting group. The two prosthesis designs had the same geometry in the coronal plane; however, the PCL-retaining design had nonconstrained AP and rotational movement, while the PCL-substituting design had only nonconstrained rotational movement. The LCS^®^ femoral component had an anatomic articulating surface, and the radii of curvature posteriorly decreased. The LCS^®^ femoral and tibial components were fully conforming in the sagittal plane from full extension to 30° flexion and less conforming for greater flexion because of the decreasing radii of curvature of the femoral posterior condyles.

One surgeon (YI) performed all surgeries using a standardized technique as previously described [16]. In all knees, the femoral components were fixed without cement, and the tibial components were fixed using cement. The patella was not resurfaced, and no lateral retinaculum release was performed in any case.

Ligament-balancing techniques, which included the necessary soft tissue release and removal of peripheral osteophytes, were used and confirmed with spacer blocks to ensure a balanced knee with equal flexion and extension gaps. The proper intraoperative coronal and sagittal plane laxity was confirmed manually, although no intraoperative quantitative evaluation was performed. For femoral sizing, we always placed the femoral template against the lateral condyle to visually determine the best fit with the knee in flexion. The femoral template used to size the femur was fitted on the lateral border of the bone surface, rather than on cartilage. When sizing, we maintained the anterior flange of the femoral component in the same plane as the anterior cortex.

Postoperative treatment included the use of a bulky compression dressing, an intraarticular drain, and the drop and dangle technique of Kumar et al. [21]. On the first postoperative day, full weightbearing, as tolerated with a cane, and exercises were allowed under the supervision of a therapist. Beginning 1 week after surgery, passive ROM exercises were performed every day. Patients received at least 2 hours of daily physical therapy, including isometric exercises, passive ROM, active-assisted ROM, quadriceps and hamstring strengthening, and gait training (including ascending and descending stairs). All patients received functional electrical stimulation.

One independent physical therapist (TM) measured flexion with a standard hand-held goniometer with 38-cm arms. The patient rested in the supine position on the table, and the physical therapist determined the maximum passive flexion under nonweightbearing conditions. The lateral femoral condyle was used as a landmark to center the goniometer. The proximal limb was directed toward the greater trochanter and the distal limb toward the lateral malleolus. The physical therapist measured and recorded the amount of knee flexion to the nearest 5°. The flexion angle was examined preoperatively and 12 months postoperatively, which provided sufficient time to predict ROM after TKA [5, 14, 27, 29, 32].

**Fig. 1A–B:**
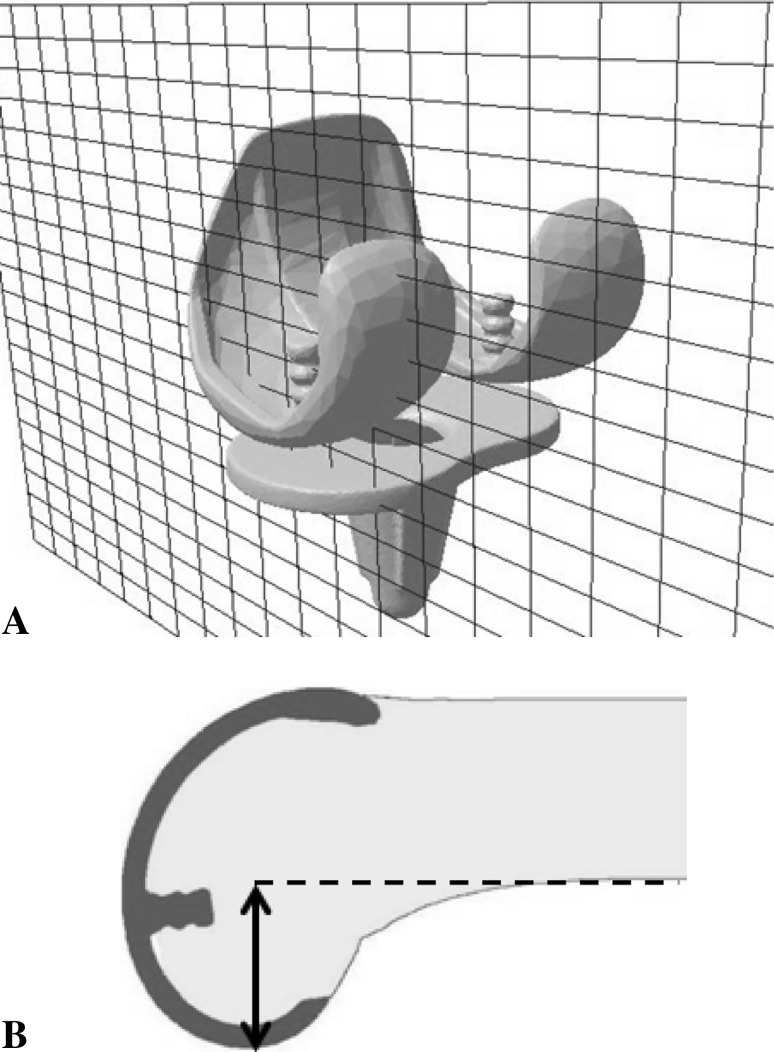
Cross-sectional views in the sagittal plane of the femoral and tibial components of a prosthesis used in TKA are shown. (**A**) A digital model of the prosthesis complex is shown. (**B**) Measurement of the maximum condyle thickness (double-headed arrow) is made from the farthest edge of the condyle to a line drawn tangent to the posterior femur shaft (dotted line).

We used a quantitative 3-D technique developed by Sato et al. [30, 31] to measure changes in the medial and lateral femoral condylar offsets. This assessment required acquisition of preoperative CT images of each patient’s femur and tibia. In addition, biplanar computed radiography images of the lower extremities were obtained before and after TKA. The biplanar computed radiography images were downloaded to a personal computer using the 3-D lower extremity alignment assessment system Knee CAS. The 3-D digital bone models and component models were projected onto the biplanar computed radiography images using the camera calibration technique. Matching the silhouettes of these digital models to the contours of the respective bone images and component computed radiography images through 3-D rotation and translation allowed computation of the 3-D position and alignment of the components relative to the femur and tibia. After these image-matching procedures, a 3-D view of the digital model complex was displayed in which the component models were implanted in the bone models. Any distance between points in the 3-D digital model could be computed, and a cross-sectional view of the 3-D digital model complex could be displayed for any plane (Fig. 1A). More detailed information about this system has been published [2, 20, 30, 31].

To minimize interobserver variation, an experienced technician (HI) performed all tests. The maximum spatial errors of this procedure were 0.5 mm when determining distance. Regarding the reproducibility of the calculated distance, we recognized the maximum intraobserver error, including all analytical processes, as 0.9 mm.

Preoperative and postoperative posterior condylar offsets were measured from the 3-D cross-sectional views in the sagittal plane for the preoperative femur and the femoral component. The maximum thicknesses of the medial and lateral posterior condyles were measured from the edge of each condyle to a line tangential to the posterior cortex of the femoral shaft (Fig. 1B).

We used Spearman’s rank correlation coefficients to evaluate relationships between changes in each posterior condylar offset and the post-TKA maximum knee flexion angle. Based on a power analysis, we estimated 85 samples would be necessary to detect a correlation coefficient of 0.3 with 80% power. The values were expressed as the mean ± 1 SD. In all tests, a p value less than 0.05 was considered significant. Statistical analyses were performed using IBM^®^ SPSS^®^ Statistics, Version 19 (IBM Japan, Inc, Tokyo, Japan).

## Results

**Fig. 2A–B:**
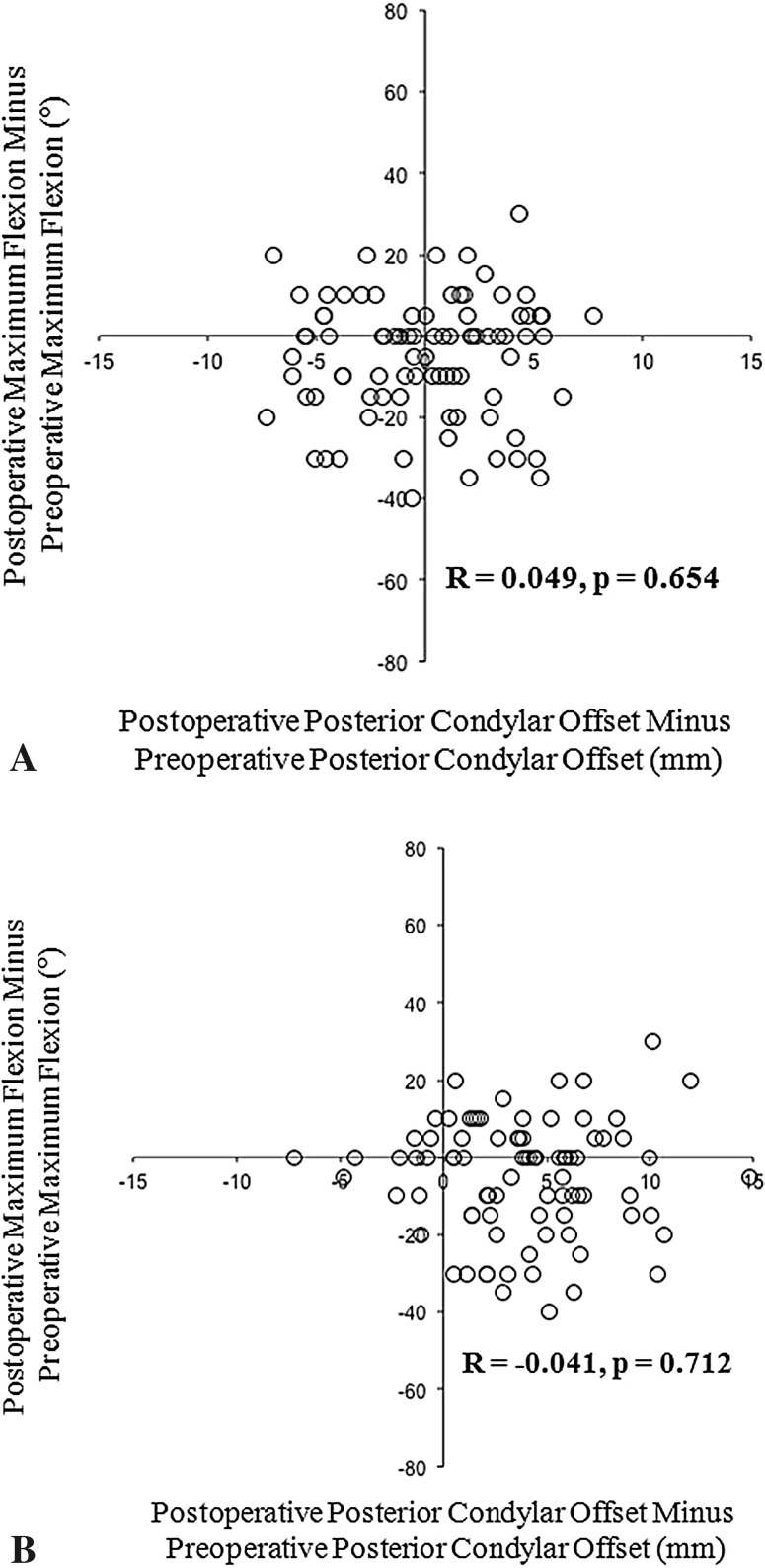
Correlations between the (**A**) medial and (**B**) lateral posterior condylar offset difference and the change in knee flexion with PCL-retaining prostheses are shown.

In PCL-retaining meniscal-bearing knees, there were no significant correlations between the changes in the posterior condylar offsets and the post-TKA knee flexion angles (post-TKA knee flexion angle versus posterior condylar offset change in medial condyle: R = 0.049, p = 0.654 [Fig. 2A]; post-TKA knee flexion angle versus posterior condylar offset change in lateral condyle: R = −0.041, p = 0.712 [Fig. 2B]). In these knees, the mean medial posterior condylar offset was 26.1 ± 2.9 mm preoperatively and 26.1 ± 3.9 mm postoperatively. The difference between preoperative and postoperative offsets for the medial condyle was 0.0 ± 3.6 mm (same or increased in 46 joints and decreased in 39 joints). The mean lateral posterior condylar offset was 25.1 ± 2.4 mm preoperatively and 28.9 ± 3.6 mm postoperatively. The difference between preoperative and postoperative offsets for the lateral condyle was 3.8 ± 3.9 mm (same or increased in 73 joints and decreased in 12 joints). The maximum knee flexion angle was 117° ± 17° preoperatively and 112° ± 15° postoperatively. The postoperative difference in maximum knee flexion angle for PCL-retaining meniscal-bearing knees was −5° ± 15°.

**Fig. 3A–B:**
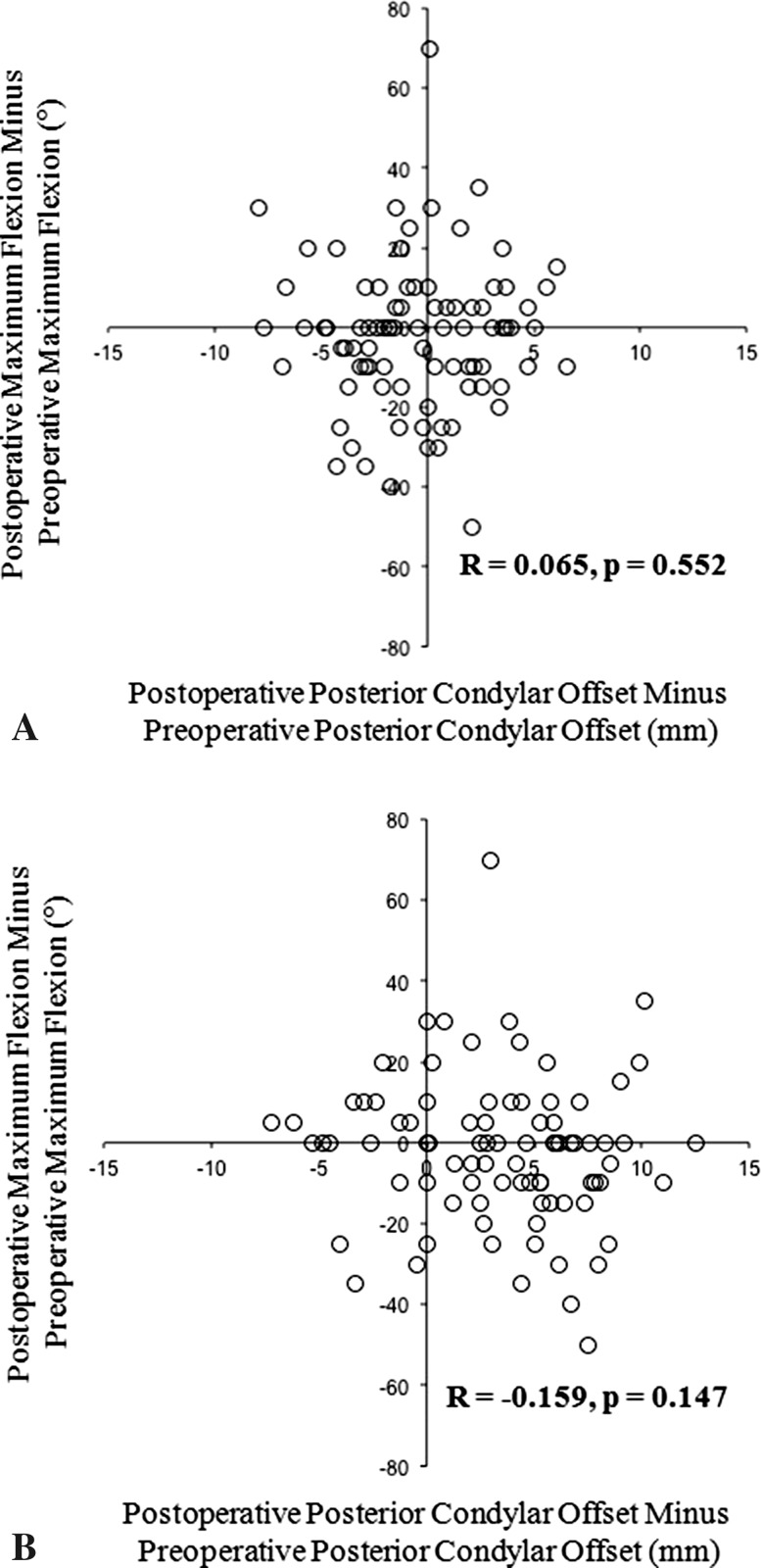
Correlations between the (**A**) medial and (**B**) lateral posterior condylar offset difference and the change in knee flexion with PCL-substituting prostheses are shown.

In PCL-substituting rotating-platform knees, there were no significant correlations between the changes in the posterior condylar offsets and the post-TKA knee flexion angles (post-TKA knee flexion angle versus posterior condylar offset change in medial condyle: R = 0.065, p = 0.552 [Fig. 3A]; post-TKA knee flexion angle versus posterior condylar offset change in lateral condyle: R = −0.159, p = 0.147 [Fig. 3B]). In these knees, the mean medial posterior condylar offset was 25.8 ± 2.4 mm preoperatively and 25.4 ± 3.5 mm postoperatively. The difference between preoperative and postoperative offsets for the medial condyle was −0.5 ± 3.3 mm (same or increased in 40 joints and decreased in 45 joints). The mean lateral posterior condylar offset was 24.8 ± 2.4 mm preoperatively and 28.2 ± 4.3 mm postoperatively. The difference between preoperative and postoperative offsets for the lateral condyle was 3.3 ± 4.2 mm (same or increased in 69 joints and decreased in 16 joints). The maximum knee flexion angle was 114° ± 20° preoperatively and 112° ± 15° postoperatively. The postoperative difference in maximum knee flexion angle for PCLS rotating-platform knees was −2° ± 18°.

## Discussion

Previous studies using plain radiographs have disagreed about whether medial or lateral femoral posterior condylar offset affect the functional outcome measure of maximum knee flexion after TKA [1, 3, 11, 12, 18, 19, 23, 24]. The most important finding of this CT-based study was that there were no statistical correlations between the changes in medial and lateral posterior condylar offsets after surgery and the changes in maximum knee flexion in one design of a mobile-bearing PCL-retaining and one design of a rotating-platform PCL-substituting prostheses.

There are four main limitations of this study. First, since this is quasirandomized design using chart numbers, the numbers of females enrolled in the study is seven times higher than males. The higher percentage of females in this series presumably is attributable to the preponderance of severe primary osteoarthritis of the knee in Japanese female patients resulting from the inherent bowleg deformity associated with the habitual Japanese sitting (squatting) position. Second, the results may not be generalized to all patients with knee arthroplasties because the study participants were patients with osteoarthritis who experienced satisfaction in activities of daily life after surgery. In addition, we evaluated only mobile-bearing designs; the current femoral prostheses have the same geometry in the sagittal plane for the medial and lateral condyles and the same constraints between the femur and tibial inserts. Careful attention should be paid to changes in conformity of femoral and tibial coupling with flexion, especially in the current prostheses as compared with those with a femoral component design having a single radius.

Third, because the current system requires that CT scanning be performed before surgery (although only once), it is more invasive in terms of radiation exposure than conventional two-dimensional (2-D) analysis. Finally, correctly evaluating the thickness of any cartilage remaining on the posterior condyles is difficult with our current system using CT and computed radiography techniques. Clarke [6] concluded that changes in the posterior condylar offset after TKA cannot be determined by radiographic measurements alone because cartilage thickness varies in posterior condylar specimens resected at TKA. The development of a new radiation-free system that more accurately evaluates cartilage thickness, such as the use of MRI to produce 3-D digital bone models, is expected [2]. Despite these limitations, strengths of the study include that the patients were treated by one experienced surgeon using the same instrumentation for all cases. Furthermore, the methods used can provide information on the correlation between each posterior condylar offset and the post-TKA knee flexion angle to overcome the limitation of 2-D analysis [16].

Many studies have described the various factors involved in knee flexion angle after TKA, including preoperative flexion angle [10, 17, 18, 29], preoperative alignment [17, 29], implant design [9, 22, 34], posterior tibial slope [4, 23, 24], anterior movement of the femur [3, 24], surgical technique [25, 29], and rehabilitation protocol [26, 28]. Considering these studies, preoperative knee flexion angle might be regarded as the most crucial factor for predicting the postoperative flexion angle. Posterior condylar offset (based on radiographic evaluation) may be an important factor, although contradictory results have been reported [1, 3, 11, 12, 18, 19, 23, 24]. Knee flexion is limited theoretically by direct impingement of the posterior aspect of the tibial component against the posterior aspect of the femur. In fact, Bellemans et al. [3] observed this fluoroscopically in 72% of their patients. They concluded that restoration of posterior condylar offset was important because it allows a greater degree of flexion before impingement occurs. However, several factors were attributable to the impingement, including posterior condylar offset, a paradoxic anterior movement with flexion, component design such as a high posterior lip on the polyethylene insert, and the degree of posterior tibial slope. The inability to maintain or restore a functional PCL is believed to be the cause of paradoxic forward sliding of the femur during flexion. This has been shown to lead to early impingement and limited flexion by videofluoroscopic studies [3, 8]. Hanratty et al. [12] used radiographic evaluations to analyze the PCL-substituting prosthesis used in the current study. In agreement with our results, they found no statistical correlations between the change in each posterior condylar offset and the changes in knee flexion 1 year after TKA.

The in vivo kinematics and knee flexion angle have been shown to be similar for current mobile-bearing PCL-retaining and PCL-substituting prostheses [33], although the PCL-retaining design has nonconstrained AP and rotational movement and the PCL-substituting design has only nonconstrained rotational movement. Both designs showed approximately 1 mm of AP movement between 0° and 90° flexion. Thus, both prosthetic designs showed almost the same positioning of the femoral component on the tibial component during knee flexion. Another in vivo kinematic study [15] of the PCL-retaining prosthesis used in the current study showed that the average movement of the medial condyle was only 1.3 mm in the anterior direction, while the lateral condyle moved 1.5 mm in the posterior direction from full extension to 90° knee flexion. From full extension to maximum knee flexion, the average amount of movement of the medial condyle was only 1.7 mm anteriorly, while the lateral condyle moved 2.6 mm posteriorly. Although we did not perform a kinematic analysis in our study, the current PCL-retaining prosthetic design, with a 10° posterior tibial slope, had less of an effect on the nonconstrained AP movement and did not significantly affect the paradoxic anterior movement with flexion as effectively as did the PCL-substituting design, which has only nonconstrained rotational movement.

We investigated the influence of medial and lateral posterior condylar offsets on maximum knee flexion after TKA using CT and a 3-D lower extremity alignment assessment system. Differences in individual posterior condylar offsets were not correlated with changes in postoperative knee flexion 1 year after TKA for the current PCL-retaining or PCL-substituting prosthetic designs. We should recognize that correctly identifying which condyle affects the results of the TKA may be difficult with conventional radiographic techniques. Additional studies are needed to research whether various patterns of change in each condyle provide significant effects on knee flexion after TKA.
